# Hepatitis C elimination in people living with HIV – the importance of biomedical and behavioural interventions

**DOI:** 10.1002/jia2.25589

**Published:** 2020-07-28

**Authors:** Daniela K van Santen, Rachel Sacks‐Davis, Margaret Hellard

**Affiliations:** ^1^ Disease Elimination Program Burnet Institute Melbourne Australia; ^2^ Department of Infectious Disease Research and Prevention Public Health Service of Amsterdam Amsterdam the Netherlands; ^3^ School of Public Health and Preventive Medicine Monash University Melbourne Australia; ^4^ Department of Infectious Diseases The Alfred and Monash University Melbourne Australia; ^5^ Doherty Institute and Melbourne School of Population and Global Health University of Melbourne Melbourne Australia

**Keywords:** HIV/HCV co‐infection, people living with HIV, elimination, direct‐acting antivirals, behaviour

Hepatitis C virus (HCV) infects an estimated 71 million people, including over two million with HIV/HCV coinfection [1]. The World Health Organization (WHO) aims to eliminate HCV as a public health threat by 2030, reducing incidence by 80% and HCV‐related mortality by 65% from 2015 numbers. People living with HIV (PLHIV) are at six times higher odds of HCV infection than their HIV‐negative counterparts; coinfected people have three times the mortality of HCV‐monoinfected individuals [1, 2], and 12 times general population mortality, so are key to elimination [3]. HIV/HCV coinfection is common within vulnerable populations, including people who inject drugs (PWID), due largely to sharing injecting equipment, but also HIV‐positive gay and bisexual men (GBM), due largely to high‐risk sexual behaviour (often combined with drug use) [1].

While many countries are struggling to meet HCV elimination targets [4], optimism about hepatitis C elimination among PLHIV is justified. Before direct‐acting antiviral (DAA) therapy became available in 2013, HCV cure rates were lower in PLHIV (<50%) than in HCV‐monoinfected individuals. Consequently, PLHIV were considered difficult to treat. Now, sustained viral response rates in PLHIV match those in HIV‐negative people, with DAAs curing >95% of chronic infections in eight to twelve weeks. Also, many PLHIV in high‐income countries are engaged in routine HIV clinical care, facilitating annual HCV testing and rapid treatment [5]. High treatment uptake was reported among PLHIV after DAAs became available, and while uptake may have declined among HCV‐monoinfected individuals [6], this might not apply to PLHIV given their greater engagement in care.

Nevertheless, maintaining HCV treatment rates in PLHIV requires vigilance; treatment uptake varies across risk groups. Even in countries with sound HIV care and high DAA uptake, linkage and retention in HIV care is typically lower among PWID and migrant populations than in GBM [7]. Moreover, in low‐and‐middle‐income countries, poor linkage and loss to follow‐up remain important bottlenecks in the HIV cascade of care, reducing opportunities for HCV diagnosis and treatment uptake. In addition, some countries restrict DAA treatment based on injecting drug use, alcohol use and stage of liver disease [4]. Hence micro‐elimination of HCV in PLHIV is more challenging in some subgroups (such as PWID) and where access to screening and treatment is restricted.

Despite the potential to eliminate HCV in PLHIV, empirical evidence of progress is scarce, with few reports in high‐income countries of reduced HCV incidence and liver‐related mortality in PLHIV compared to pre‐DAA levels [8, 9]. These studies had only short follow‐up after DAAs became available, and lacked matched calendar‐time control groups, weakening attribution of outcomes to DAAs. For example soon after DAA introduction in the Netherlands, researchers reported a 51% reduction in HCV incidence in GBM living with HIV across this period [8]. However recent data from the Netherlands suggests that HCV incidence was declining already, making it difficult to attribute the decline, in part or in full, to DAAs [10]. Moreover, while the prevention impact of HIV treatment on incident infections (treatment as prevention) has been widely studied, no such data exist for HCV. Studies of the population‐level effect of DAA therapy on incidence and mortality are needed to evaluate whether elimination is feasible with treatment alone or requires additional interventions. However, studies with time‐matched controls are challenging, there is insufficient proximity of liver‐related mortality to cohort enrolment, and key events such as reinfection, that may impact on outcomes, are uncommon in individual cohorts. Obtaining clear evidence that HCV elimination is feasible in PLHIV requires combining datasets and using innovative methodological approaches, such as quasi‐experimental study designs.

Prevention remains a pillar of HCV elimination, because treatment alone is unlikely to eliminate HCV in many settings due to ongoing high‐risk behaviours. Combined needle and syringe programmes and opioid agonist therapy reduce HCV risk by 85% among PWID [11], but low harm reduction (HR) programme coverage in most countries threatens HCV elimination. In the United States, where the opioid crisis drives HCV transmission, elimination in PWID will fail without HR programme scale‐up [12]. Moreover, to maximize uptake and impact, PWID must access to low threshold HR programmes which allow them to enter, exit and re‐enter HR programmes without restrictions on their drug use. Almost no new HCV infections have been observed among PWID in Amsterdam over the past two decades, partly due to early adoption of low‐threshold HR programmes [11]. The Netherlands’ pragmatic HR approach represents a blueprint for programme implementation, service delivery and practice. HR programmes also serve to bring PWID and health care professionals together, providing valuable opportunities for engagement in HCV testing and care.

While HR programmes are proven interventions for PWID, evidence of the effectiveness of behavioural interventions in preventing HCV in GBM is scarce. This presents a considerable challenge, because based on modelling, HCV elimination targets will be missed in this group without behavioural interventions [13]. Another key challenge is the intersection of sexual and drug use risks, and ongoing uncertainty over which behaviours are most important. Because these behaviours are so highly correlated and cohorts with detailed longitudinal data are small, no study has disentangled their effects. Standard risk‐factor association‐based analysis cannot distinguish whether non‐injecting drug use is a transmission pathway for HCV or whether sexual practices drive transmission. Pooling behavioural data could improve understanding of the causal pathways of HCV transmission in GBM living with HIV, enable development of tailored strategies to prevent new infections, and provide evidence to refine current prevention activities.

In addition to behavioural interventions, ongoing and systematic (post‐treatment) HCV testing is essential due to ongoing risk behaviour in both PWID and GBM living with HIV. Reinfection rates among PLHIV span 1–15/100 person‐years (PY), and up to 38/100 PY for subsequent reinfections in GBM living with HIV [14, 15]. While the available evidence suggests primary HCV incidence is declining [10], recent studies across the DAA threshold suggest that reinfection rates have not diminished, but their scarcity and short follow‐up prevent definitive conclusions [14]. A Swiss HIV/HCV coinfection model of the potential epidemic effects of DAA scale‐up predicted that reinfections will increase as a proportion of incident infections among GBM living with HIV (e.g. 44% of all incident infections in 2030, assuming increased risk behaviour) [13]. Whilst appearing counterintuitive, detection of high rates of reinfection among GBM living with HIV following HCV treatment, particularly where treatment uptake has been high, suggests that those at risk of onward transmission are being treated and tested, which is important for reducing HCV incidence. This highlights the crucial role of ongoing monitoring of HCV RNA and prevention after treatment in those with ongoing risk behaviour.

In many countries, undiagnosed HCV infections in PLHIV are another challenge. While some high‐income countries report few undiagnosed HIV infections, and high proportions of diagnosed PLHIV ever tested for HCV, most countries are performing insufficient testing to facilitate the treatment uptake required to reach WHO elimination targets [4]. Reaching and treating all PLHIV might involve testing at pharmacies, peer‐based testing, home‐based antibody testing and RNA testing using dried blood spots. Importantly, little is known about trends in HCV testing uptake and whether HCV testing guidelines are being followed in PLHIV care.

Achieving HCV elimination in PLHIV requires further empirical evidence about the population‐level effectiveness of DAA treatment and behavioural interventions among GBM living with HIV to reduce incidence and mortality. To progress meaningfully towards elimination, we also need to fully utilize available HCV prevention tools and strategies for increasing testing and treatment uptake. This includes expanding HR programmes and community‐based testing and treatment, and ensuring universal access to affordable DAAs.

## COMPETING INTERESTS

Dr Hellard has received investigator‐initiated research grants from Gilead Sciences, AbbVie and Bristol‐Myers Squibb, unrelated to the submitted work. Dr Sacks‐Davis and Dr van Santen have nothing to disclose.

## AUTHORS’ CONTRIBUTIONS

DVS, RSD and MH wrote the manuscript together.
